# Comprehensive high-resolution multiple-reaction monitoring mass spectrometry for targeted eicosanoid assays

**DOI:** 10.1038/sdata.2018.167

**Published:** 2018-08-21

**Authors:** Carlos Artério Sorgi, Ana Paula Ferranti Peti, Tania Petta, Alyne Fávero Galvão Meirelles, Caroline Fontanari, Luiz Alberto Beraldo de Moraes, Lúcia Helena Faccioli

**Affiliations:** 1 Departamento de Análises Clínicas, Toxicológicas e Bromatológicas, Faculdade de Ciências Farmacêuticas de Ribeirão Preto, Universidade de São Paulo, Av. do Café, s/n, Ribeirão Preto, São Paulo 14040-903, Brazil; 2 Departamento de Química, Faculdade de Filosofia, Ciências e Letras de Ribeirão Preto, Universidade de São Paulo, Av. Bandeirantes, 3900, Ribeirão Preto, São Paulo 14049-901, Brazil

**Keywords:** Mass spectrometry, Lipidomics

## Abstract

Eicosanoids comprise a class of bioactive lipids derived from a unique group of essential fatty acids that mediate a variety of important physiological functions. Owing to the structural diversity of these lipids, their analysis in biological samples is often a major challenge. Advancements in mass spectrometric have been helpful for the characterization and quantification of these molecular lipid species in complex matrices. However, there are technical limitations to this approach, including low-abundant and/or poorly ionizable lipids. Using high-resolution multiple-reaction monitoring (MRM^HR^), we were able to develop a targeted bioanalytical method for eicosanoid quantification. For this, we optimized the LC-MS/MS conditions and evaluated several parameters, including linearity, limits of quantification, matrix effects and recovery yields. For validation purposes, we looked at the method’s precision and accuracy. A library of high-resolution fragmentation spectra for eicosanoids was developed. Our comprehensive dataset meets benchmark standards for targeted analysis, having been derived using best-practice workflows and rigorous quality assessments. As such, our method has applications for determining complex eicosanoid profiles in the biomedical field.

## Background & Summary

**Figure 1:**
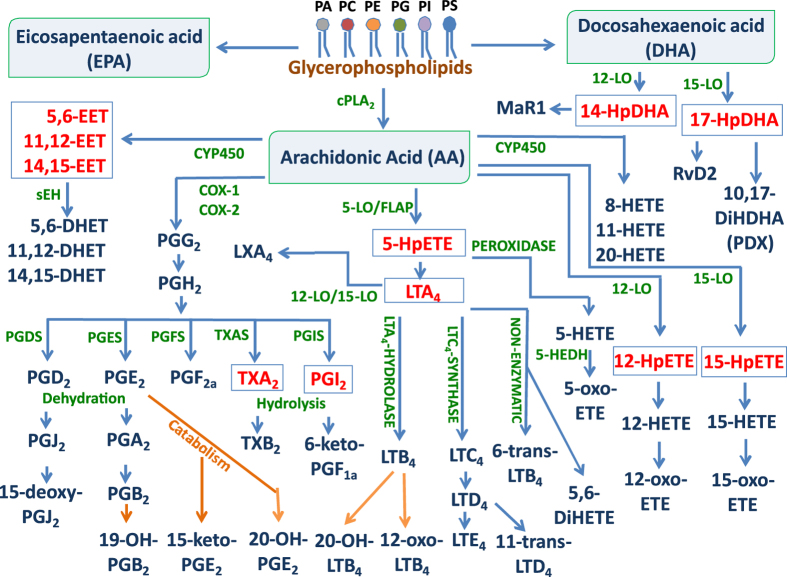
Schematic outline of metabolites derivate from arachidonic acid (AA-20:4) via the lipoxygenase (LOX), cyclooxygenase (COX), CYP450 (CYP) or free radical catalyzed pathways. The enzymes involved in eicosanoid biosynthesis were in green and orange arrows indicated the catabolism pathway. Major metabolites (Blue) derived from those metabolic pathways are included in the MRM^HR^ quantitation assay. The non-stable intermediated metabolites (Red) were not included in the method.

Lipidomics involves the identification and quantification of lipids within a given biological system^1^. Recent advances have been enabled by the development of new mass spectrometric tools and protocols for the analysis of molecular lipids in complex matrices^2^. Polyunsaturated fatty acids (PUFAs) are known to mediate some of their bioactivities through the formation of oxygenated metabolites. These bioactive lipids include eicosanoids, a class of lipid mediators derived from arachidonic acid via cyclooxygenase (COX), lipoxygenase (LOX), and cytochrome-P450-catalyzed reactions (CYP450), and through non-enzymatic lipid peroxidation^3^ (Fig. 1). The oxygenated lipid mediators play prominent roles in the physiological and pathological regulation of many biological processes, including those observed in inflammatory diseases^3^.

Using targeted, as opposed to global, lipid analysis provides an alternative method in lipidomics^4^. The analysis of lipid mediators in biological samples has its own set of challenges. This is because these lipids are often present in extremely low concentration, are transiently formed on demand by cells, and often have limited half-lives. To compound the problems faced by researchers, many isomeric species can be generated, which also have a specific metabolic function^5^. Therefore, highly sensitive and accurate methods are urgently needed for the analysis of eicosanoids^6^.

A wide range of techniques has been used for the separation, detection, and quantification of lipid mediators. Currently, progress in liquid-chromatography–electrospray ionization tandem mass spectrometry (LC–ESI-MS/MS) offers a powerful approach for this purpose^7^. The advantage of mass spectrometry is its capacity to separate and characterize ionized analytes according to their mass-to-charge ratios (*m/z*). Collision-activated dissociation (CAD) can be used to determine structural information acquired through the lipid’s ion fragmentation^8^. LC-MS/MS methods for targeted lipidomics utilize scheduled multiple-reaction monitoring (MRM) to optimize sensitivity, maximize the number of metabolites that can be analyzed at any given time, and reduce the time required for analysis^9^. The beauty of LC is that it simplifies analysis and offers the greatest chromatographic advantage for separating complex mixtures of lipid mediators because no prior derivatization of the analytes was required^6^. Despite this, chemical similarities and the presence of isomeric species still make separating lipid mediator species via LC rather difficult.

MS detection of the biological molecules was found to be dependent on ESI for ion generation, which produced both positive and negative molecular ion species ([M+H]^+^ and [M-H]^−^)^10^, even though our analysis was conducted in the negative-ion mode. Tandem mass spectrometry (MS/MS) is known to increase the sensitivity and specificity of quantitative lipid analyses^1^. Since the acquisition mode MRM in most lipidomics studies is used to monitor the analytes through the transition of a select precursor ion to a specific product ion^4^, there are several lists which feature characteristic eicosanoids MRM transitions performed on triple-quadrupole instruments^6,11^.

**Figure 2:**
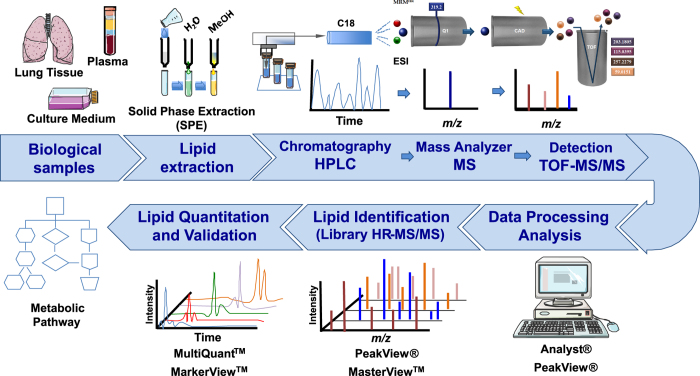
Experimental workflow of lipid mediators analysis using MRM^HR^. Various biological samples, such as lung tissue, plasma and culture medium, were prepared by solid phase extraction (SPE) for subsequent UHPLC-MS/MS analysis. The scheme of MRM^HR^ analysis was represented by 5-HETE detection (precursor ion at *m/z* 319.2 and fragment ions at *m/z* 257.2279, 203.1805, 115.0395 and 59.0151) in a complex mixture. The lipids were identified and quantified using bioinformatics software package. A library of HR-MS/MS profile was created for each metabolite quantified. A partial validation of MRM^HR^ method was conducted according FDA recommendations.

In order to gain greater confidence in detection and quantification methods, the standard practice follow selecting two or more transitions per precursor ion for analysis^12^. Modern instruments have the required speed to cycle through many precursor ions with multiple transitions within a short time window^13^. Indeed, as an alternative to the classical MRM performed on triple-quadrupole, hybrid systems like the TripleTOF5600^+^, a quadrupole time-of-flight (QTOF) instrument can be used to acquire high-resolution MRM (MRM^HR^)^13^. In a MRM^HR^ assays, the first quadrupole basically selected the precursor ion passing into the collision cell, and the instrument subsequently monitored all transitions of this precursor ion. The TOF analyzer recorded the resultant high-resolution spectrum. If retention times were known prior to analysis, many precursors would be selected during a scheduled MRM^HR^ experiment; providing higher confidence detection, since all possible transitions can be monitored^13^. Indeed, targeted eicosanoid lipidomics could be performed using a comprehensive MRM^HR^ strategy, which included important analytical steps, such as the standardization of the operating procedures for sample collection and preparation, as well as the development of both the LC-MS/MS method and the computational process^12^ (Fig. 2).

Herein, detailed descriptions of LC optimization of target eicosanoid separation, MRM^HR^ parameters, and method validation for quantification in biological sources were provided. Additionally, a new library of eicosanoid fragmentation in high resolution was reported. As demonstrated in our recent work in the field of macrophage biology^14^, this MRM^HR^ method was found to be useful for comprehensive investigations on pathway elucidation during numerous biological processes.

## Methods

Descriptions for the procedures were taken from our previous work^14^ and were either completely adapted or supplemented with new details where necessary.

### Reagents

All eicosanoids, fatty acids (molecular weight standards, MWS), and deuterated internal standards (IS) were purchased from Cayman Chemical Co (Ann Arbor, MI, USA). The details of all analytical-grade standards are listed in file “Metabolite standard details (docx) and Metabolite standard details (pdf), Data Citation 1”. HPLC-grade solvents acetonitrile (ACN), methanol (MeOH), isopropanol were purchased from Merck (Kenilworth, NJ, USA), and deionized water (H_2_O) were obtained from Milli-Q integral water purification System for ultrapure water from Merck-Millipore (Kenilworth, NJ, USA). The acetic acid and ammonium hydroxide (NH_4_OH) used in these experiments came from Sigma-Aldrich (St. Louis, MO, USA).

### Animals

Six-week-old male C57BL/6 mice were obtained from *Faculdade de Ciências Farmacêuticas de Ribeirão Preto*, FCFRP/USP. All animal experiment protocols were in compliance with institutional guidelines for ethics in animal experiments approved by the Animal Care Committee of the *Universidade de São Paulo* (Permit no. 11.1.468.53.6). Euthanasia protocols were performed under CO_2_/O_2_ excess atmosphere. Furthermore, all efforts were made to minimize suffering.

### Sample preparation

*Standard Solutions and Calibration Curve:* Individual stock solutions of lipid mediators (MWS) and IS were prepared at 10 μg.mL^−1^ in MeOH. A standard working solution (WS) composed of a mixture of all of the lipids (MWS and IS) was prepared via direct infusion of 100 ng.mL^−1^ in MeOH/H_2_O (7:3, *v/v*) containing 0.1% NH_4_OH by volume; this was to establish the MRM^HR^ parameters, such as the declustering potential (DP) and the collision energy (CE). The WS was prepared without adding any isomer compounds to the same solution. For LC parameter studies, the WS was prepared as described with no addition of NH_4_OH. In another set of experiments, the calibration curves for the validation assay were obtained by diluting the relevant lipids in MeOH/H_2_O (7:3, *v/v*) to the following final concentrations: 2.3, 4.6, 9.2, 18.5, 37.0, 74.0, 148.1, 222.2, 333.3, and 500 ng.mL^−1^. To determine matrix effect and recovery yields, IS solutions were prepared in MeOH at three concentrations 18.5, 148.1, and 500 ng.mL^−1^. All solutions were stored in amber glass vials at −80°C and under argonium atmosphere until needed.

*Solid Phase Extraction (SPE):* Pooled plasma and lung tissue samples were obtained from C57BL/6 mice. Dulbecco’s Modified Eagle Medium (DMEM, Gibco-Grand Island, NY, USA) was used as the culture medium for the validation assay. Lipid extraction was carried out according to a previously described protocol^15,16^, but with slight modifications. In brief, each sample was spiked with 10 μL of the IS solution before being extracted for use either in the recovery assay or the matrix effect assay. Lung samples were prepared via homogenization (Mixer Homogenizer-Labortechnik, Staufen, Germany) of 50 mg of tissue per mL of MeOH/H_2_O (1:1, *v/v*) solution. Plasma samples were made via protein precipitation of 150 μL of the sample in 1.5 mL of MeOH/ACN (1:1, *v/v*) at 4 °C, which was left to denature overnight. Afterward, both plasma and lung samples were centrifuged for 10 min at 4 °C and 800 x *g*. For culture medium extraction a solution in MeOH was preparade (1:1, *v/v*). Subsequently, the supernatant samples, from lungs, plasma or culture medium were diluted with water to a maximum solvent concentration of 15%.

For the first step in the SPE extraction protocol, the cartridge (Hypersep C18-500 mg, 3 mL, Thermo Scientific-Bellefonte, PA, USA) was washed with 4 mL of MeOH and equilibrated with 4 mL of H_2_O that had been generated using an extraction manifold (Waters-Milford, MA, USA). After loading the diluted samples, the cartridges were again flushed with 4 mL of H_2_O to remove hydrophilic impurities. The analytes which had been adsorbed on the SPE sorbent were eluted with 1 mL of MeOH and stored at −80 °C to prevent metabolite degradation. The solvent was removed *in vacuo* (Concentrator Plus, Eppendorf, Germany) at room temperature and re-dissolved in 50 μL of MeOH/H_2_O (7:3, *v/v*) for LC-MS/MS analysis.

### LC-MS/MS analysis

The Ultra-High-Performance Liquid Chromatography (UHPLC) system (Nexera X_2_, Shimadzu-Kyoto, HO, Japan) was equipped with a binary pump system, a SIL-30AC autosampler, a DGU-20A degasser and a CBM-20A controller. The conditions used for chromatography are outlined in a previously published work^16,17^. However, some modifications have been implemented to improve the eicosanoids quantifications. For this propose, the method was optimized using an Ascentis Express C18 column (Supelco - St. Louis, MO, USA) with an i.d. of 100 ×4.6 mm and a particle size of 2.7 μm. Elution was conducted under a binary gradient system which consisted of Phase A, H_2_O/ACN/acetic acid (69.98:30:0.02, *v/v/v*) at pH 5.8 (adjusted with NH_4_OH), and Phase B, an ACN/isopropanol (70:30, *v/v*). Gradient elution was carried out for 25 min at a flow rate of 0.6 mL.min^−1^. Gradient conditions were as follows: 0 to 2.0 min, 0% B; 2.0 to 5.0 min, 15% B; 5.0 to 8.0 min, 20% B; 8.0 to 11.0 min, 35% B; 11.0 to 15.0 min, 70% B; and 15.0 to 19 min, 100% B. At 19.0 min, the gradient returned to the initial condition of 0% B, and the column was re-equilibrated until 25.0 min. Over the course of the analyses, the column was kept at 25 °C, and the samples were maintained at 4°C in the autosampler. A 10 μL aliquot of each sample was injected onto the column. The pH of the mobile phase A was optimized to achieve the best sensitivity and chromatographic separation of lipid metabolites. As such, the pH values of phase A throughout the experiment were 3.8, 5.8, and 6.4.

The UHPLC system was interfaced with a TripleTOF5600^+^ Mass Spectrometer (Sciex-Foster, CA, USA) equipped with a Turbo-V IonSpray. An Atmospheric-Pressure Chemical Ionization probe (APCI) was used for external calibrations of the Calibrant Delivery System (CDS). Automatic mass calibration (<2 ppm) was performed using APCI Negative Calibration Solution (Sciex-Foster, CA, USA) injected via direct infusion at 300 μL.min^−1^ flow rate; this was done periodically after each of the five sample injections. An Electrospray Ionization (ESI) source in the negative ion mode was utilized for MRM^HR^ scanning. For optimization of MRM^HR^ channel settings, CE and DP values were determined for each analyte via direct infusion of MWS and IS solutions at a flow rate of 10 μL.min^−1^. Compounds were fragmented via CAD using nitrogen as the collision gas. Additional instrumental parameters were as follows: nebulizer gas (GS1), 50 psi; turbo-gas (GS2), 50 psi; curtain gas (CUR), 25 psi; electrospray voltage (ISVF), −4.0 kV; and turbo ion spray source temperature, 550°C. The mass range of the product ion experiments was from *m/z* 50 to 700, the dwell time was 10 ms, and a mass resolution of 35,000 was achieved at *m/z* 400. Data acquisitions were performed using Analyst Software (Sciex-Foster, CA, USA).

### Data processing

Identification of the lipid species obtained in the LC-MS analysis was performed using PeakView 2.1 (Sciex-Foster, CA, USA). Briefly, this software combines information in three dimensions to give a representation of the retention time, signal intensity, and *m/z* value for each analyte. PeakView software is a stand-alone application for the qualitative review of LC-MS data files. In addition to its general application in exploring and interpreting qualitative data, the software also provides the tools for analyzing accurate and nominal mass data, structures, and multiple samples at any given time. Data processing usually proceeds through multiple steps, including filtering, feature detection, alignment, and normalization. Feature detection is conducted to identify all signals caused by true ions and to avoid the detection of false positives, thereby interpolating theoretical information from the molecular data file and experimental fragments. In our study, alignment to confirm retention time differences between runs was conducted through a comparison of the IS chromatography peaks. Normalization steps help in removing factors, which cause unwanted systematic bias and can be used to determine extraction efficiency during sample preparation via ion intensities and/or peak area between measurements. This was performed by extrapolation of the area ratio among the IS and biological analyte peaks.

For quantitative analysis, we used MultiQuant. This software uses robust integration algorithms, such as MQ4 and advanced SignalFinder, and is able to generate reliable integration results faster with less user intervention in order to maximize productivity. The use of reference standards for each of the target lipid species or class was important in quantifying the lipids under investigation. In this field, it is common practice to normalize the individual molecular ion-peak intensities using an internal standard for each lipid class. The calculated ratio of analyte to internal standard is then multiplied by the concentration of said internal standard so as to obtain the concentration of a particular analyte present in the sample. To quantify the lipid mediators in our work, MRM^HR^ channels were created during computational data processing. These were instructed to select product ions of higher intensity and selectivity in order to achieve greater analytical sensitivity and avoid cross-identification. The MRM^HR^ optimized parameters used in LC-MS/MS analysis are presented in Table 1 (available online only). The final eicosanoid concentration in biological samples was usually normalized by volume, tissue weight, cell number or protein/DNA concentration.

### Validation parameters

The method was partially validated in accordance with Food and Drug Administration (FDA) recommendations^18^. The parameters of interest were the analyte’s linearity, the lower-limits of quantification (LLOQ), inter- and intraday precision and accuracy, recovery and the matrix effects in the culture medium and both the plasma and lung tissue samples.

#### Linearity and LLOQ

These were assessed by means of 10-point standard curves prepared for five replicate samples within a linear range (Table 2). Standard curves (linear regression, *1/x* weighted) were constructed by plotting the standard area against its nominal concentration (File: ‘Retention time for lipid mediators panel under different UHPLC-MS/MS chromatography conditions (pdf)’, Data Citation 1). The acceptance criterion for the coefficient of variation (CV) was ≤20% for the LLOQ level and ≤15% for levels above the LLOQ. The LLOQ was defined as the lowest concentration level at which the signal-to-noise ratio (S/N) is ≥10 and the CV is ≤20%.

#### Inter- and Intraday Precision and Accuracy

These were evaluated at three concentration levels for the quality control samples (QC): 0.15 ng.mL^−1^ for low-concentration quality control (LQC) samples; 1.5 ng.mL^−1^ for medium-concentration quality control (MQC) samples; and 5 ng.mL^−1^ for high-concentration quality control (HQC) samples. Precision and accuracy were expressed in percentage as the Relative Standard Deviation (RSD) and the Relative Error (RE), respectively. Accuracy was defined as the difference between the expected concentration (C_ex_) and the average measured concentration (C_av_) as calculated by the formula (C_ex_−C_av_/C_ex_)×100. The precision, RSD, was calculated using the formula (SD/C_av_)×100. The method was only considered to be precise if the RSD was ≤15% (≤20% for LLOQ) and accurate if the RE of the analyte concentration measured was ≤15% of the expected value (≤20% for LLOQ). The intraday precision (repeatability) and accuracy were established for five replicates for the LQC, MQC, and HQC over the course of the same day using the same calibration curve. For the interday analysis (reproducibility), the assay was conducted for three consecutive days. Interday accuracy and precision were assessed by running analysis for five calibration curves in quintuplicate over consecutive days. According to FDA guidelines, the conventionally accepted range of inter- and intraday accuracy is from 80 to 120%. Accuracy and precision results for the lipid mediators are given in Table 3 (available online only) and respectively full results on file ‘Values obtained for each replicate of quality control (HQC, MQC and LQC) assay (xlsx) and Values obtained for each replicate of quality control (HQC, MQC and LQC) assay (pdf)’, Data Citation 1.

#### Recovery and Matrix Effects

To determine the extraction efficiency of SPE for the analytes in the biological matrix studied, recovery was assessed by means of the IS solutions. Plasma and lung tissue samples, as well as the culture medium, were spiked with IS solution before SPE extraction. Recovery was determined by comparing the analytical response of the IS for the extracted samples at three concentrations (LQC, MQC, and HQC) to the IS that had not been extracted and that had been prepared in solvent (MeOH/H_2_O, 7:3, *v/v*). Recovery was expressed as the percentage of the expected value, as calculated by the average area ratio between the extraction samples and the standard samples. The extent of recovery of the IS should be consistent, precise, and reproducible. Matrix-dependent recovery was established using different matrices, such as lung tissue homogenate, plasma, and culture medium; these were spiked with IS at three concentrations (LQC, MQC, and HQC) after SPE extraction. The peak areas after extraction were compared with the corresponding peak areas of the pure IS solutions. Samples for matrix effect and recovery studies were prepared in quintuplicate, and a CV of ≤15% was used as the accepted criteria for quality control. The recovery and matrix effect percentages for lung tissue, plasma, and culture cell are shown in Tables 4, 5 and 6, and respectively full results on file ‘Values obtained for each replicate of matrix effects and recovery samples (lung tissue, plasma and culture medium) (xlsx) and Values obtained for each replicate of matrix effects and recovery samples (lung tissue, plasma and culture medium) (pdf)’, Data Citation 1.

## Data records

**Data Record 1**–(Metabolite standard details (docx) and Metabolite standard details (pdf), Data Citation 1) gives for each metabolite standard purchase from Cayman Chemical all details, such as molecular weight, molecular formula, CAS number and supplied solution, as well biological information, such as fatty acid precursor, biosynthetic pathway and chemical class. The file include Internal lipid standards, with matching natural lipid (MWS), used in for extraction control, matrix effect and recovery experiments, also standards for calibration curves (MWS) in UHPLC-MS/MS and for quantitative calculation of lipid species.

**Data Record 2**–(Tandem mass spectral libraries in high resolution (HR-MS/MS) for eicosanoid panel (pdf), Data Citation 1), described tandem mass spectral libraries in high resolution (HR-MS/MS) for eicosanoid panel. Also, the file provided information about collision energy, molecular formula and common name, as well theoretical mass-to-charge ratio (*m/z*) of precursors and fragments ions (illustrated by chemical structures). Experimental spectrum showed numerical values that correspond to the *m/z* (x-axis) and relative intensity (y-axis) for each detected metabolite. The precursors (red) and fragments (blue) ions used for quantification analysis were highlight in the spectra.

**Data Record 3**–(Retention time for lipid mediators panel under different UHPLC-MS/MS chromatography conditions (pdf), Data Citation 1) demonstrated the retention time for lipid mediators panel under different UHPLC-MS/MS chromatography conditions. The chromatography parameters differ by mobile phase pH (3.8, 5.8 and 6.4). Using a elution method of UHPLC with a reversed-phase column (C18), a mixture of commercially lipid mediators standards elutes according to their polarity, number of double bonds and chain length, allowing the separation of most isomeric and isobaric species (represented by blue and red lines respectively) dependent of pH and according to identifying fragmentation patterns described in file ‘Tandem mass spectral libraries in high resolution (HR-MS/MS) for eicosanoid panel (pdf), Data Citation 1). An asterisk represented the non-detect metabolites.

**Data Record 4**–(Linear regression equation, correlation coefficient (r2) and linear range of concentration for each analyte (xlsx) and Linear regression equation, correlation coefficient (r2) and linear range of concentration for each analyte (pdf), Data Citation 1), showed the linear regression graphics. MS files were processed for each metabolite quantified in MRM^HR^ method with mobile phase pH 5.8. The graphics describe the linear regression equation, correlation coefficient (r^2^) and linear range of concentration for each analyte assessed by means of 10-point standard curves prepared for five replicate samples. Also the retention time were described in this file.

**Data Record 5**–(Values obtained for each replicate of quality control (HQC, MQC and LQC) assay (xlsx) and Values obtained for each replicate of quality control (HQC, MQC and LQC) assay (pdf), Data Citation 1) presented all values obtained for each replicate of quality control (HQC, MQC and LQC) assay. The individual values from quality control assay were used to determine the average (*n*=5) of precision and accuracy of the MRM^HR^ method presented in Table 3 (available online only).

**Data Record 6**–(Values obtained for each replicate of matrix effects and recovery samples (lung tissue, plasma and culture medium) (xlsx) and Values obtained for each replicate of matrix effects and recovery samples (lung tissue, plasma and culture medium) (pdf), Data Citation 1) demonstrated all values obtained for each replicate of matrix effects and recovery samples (lung tissue, plasma and culture medium). In these experiments the analytes corresponded to internal standards (IS- deutered compounds) for eliminate the interference of matrix endogenous metabolites. The individual values were used to determine the average (*n*=6) of matrix effect and recovery of the extraction method presented in Tables 4, 5 and 6.

**Data Record 7**–All data files from TripleTOF^®^5600^+^ (.wiff) have been deposited to the MetaboLights metabolomics repository^19^ (Data Citation 1). Instrument RAW data files (121 files) contain total ion current, spectra and metadata for each replicate. This data set was used to provide the linear range of concentration, retention time, LLOQ (summarized in file ‘Linear regression equation, correlation coefficient (r2) and linear range of concentration for each analyte (xlsx) and Linear regression equation, correlation coefficient (r2) and linear range of concentration for each analyte (pdf), Data Citation 1), intra- and interday precision and accuracy (summarized in file ‘Values obtained for each replicate of quality control (HQC, MQC and LQC) assay (xlsx) and Values obtained for each replicate of quality control (HQC, MQC and LQC) assay (pdf)’, Data Citation 1). Also, the MTBLS641 archive (.zip) contains others files: ‘i_Investigation.txt’ that provided summary information and project descriptors; ‘s_Study.txt’ with describes the metadata related to the samples; and ‘a_study_id_metabolite_profiling_mass_spectrometry.txt’, ‘a_study_id_metabolite_profiling_mass_spectrometry-1.txt’ (Data Citation 2) which afford the contextual data for the MS analyses from data acquisition through to the multiple processing steps. All raw data files were uploaded using an ISA-tab format.

## Technical validation

### MRM^HR^ channel determination and selectivity

Technical validation of the UHPLC-MS/MS method for the comprehensive analysis of the lipid mediators on a high-resolution mass spectrometer (TripleTOF5600^+^ system) in MRM^HR^ mode was carried out before biological sample measurements were taken; this was previously described in more detail^14^. To this end, the pre-defined MRM transitions (pairs of precursor/fragment ions) in a low-resolution mass spectrometer typically used for routine lipidomics analysis in our lab^16,17^ were optimized for high-resolution mass spectra (“Tandem mass spectral libraries in high resolution (HR-MS/MS) for eicosanoid panel (pdf)”, Data Citation 1). The processing software, MultiQuant, was capable of generating these MRM-like channels post-acquisition, thus allowing that each transition was create in accordance with the MS/MS data obtained during analysis. This meant that there was no need for pre-selection of the candidate lipid fragments in the mass analyzer. In addition, time-resolved mass measurements enabled proper alignment of the detected fragment ions with their respective precursor ions for accurate identification. Table 1 (available online only) shows the optimized mass spectrometric parameters for the detection of compounds in the MRM^HR^ mode, which were done using the most sensitive and most selective MRM^HR^ transitions. These parameters allowed us to accurately discern between the individual lipid mediators and the background interferences for each analyte.

### Influence of pH on elution behavior

Developing an LC-MS/MS methodology that would efficiently assay all of the target analytes was filled with challenges, especially when it came to finding the right balance between the method’s sensitivity and chromatographic resolution. To compound matters, chromatographic separation was required for isomers that were not differentiated through MRM^HR^ transitions (i.e., those which had identical CAD spectra); these included the pairs of lipids PGE_2_/PGD_2_, LTD_4_/*trans*-LTD_4_, and LTB_4_/*trans*-LTB_4_. This observation forced us to look at the effect of pH on the elution behavior of the analytes.

**Figure 3:**
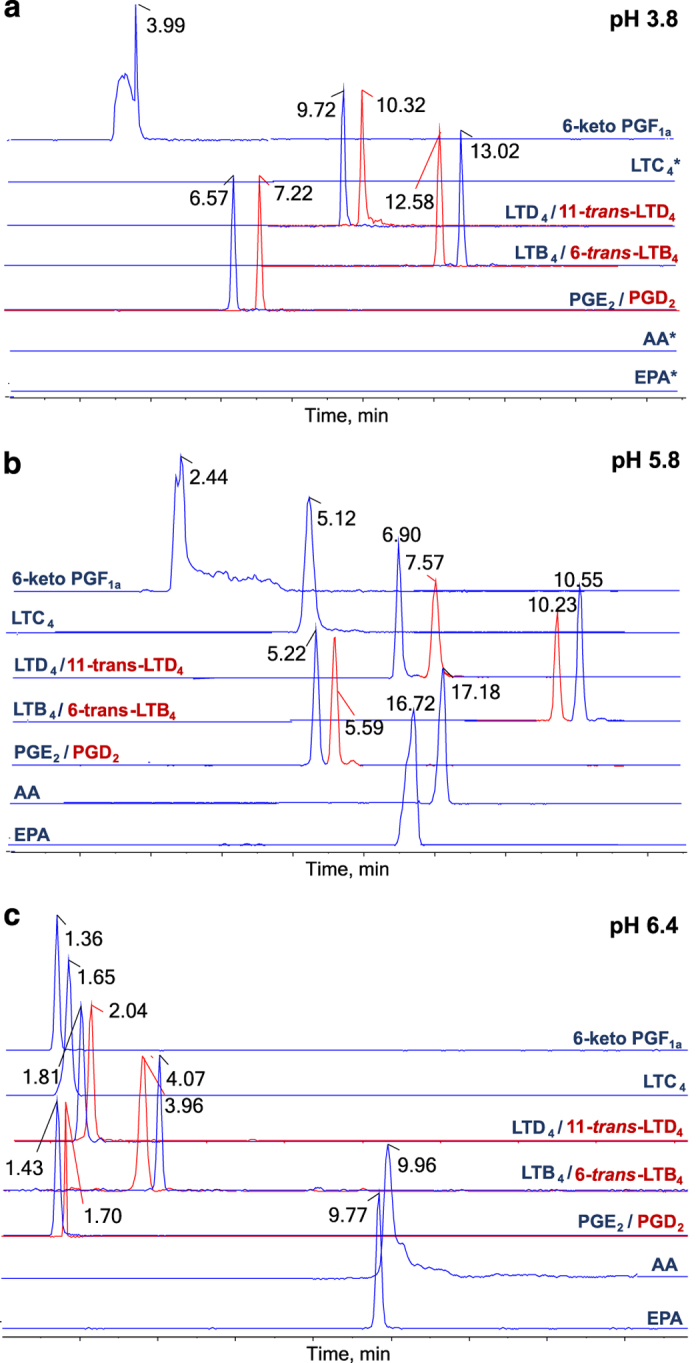
Representative MRM^HR^ eicosanoid chromatograms. A mixture of commercially available eicosanoid standards elutes in C18 column according to different experimental conditions of phase A elution: (**a**) pH 3.8, (**b**) pH 5.8 and (**c**) pH 6.4. The chromatograms showing separation of pairs of isobaric species with identical fragmentation patterns (LTD_4_ and 11-*trans*-LTD_4_, PGE_2_ and PGD_2_, LTB_4_ and 6-*trans*-LTB_4_), also showing elution behavior of critical metabolites (LTC_4_, 6-keto-PGF_1α_, EPA, and AA). The absence of metabolites peaks was represented by an asterisk (*). The retention time of all lipid mediators quantified in MRM^HR^ method, for different pH elution conditions, were available in file ‘Retention time for lipid mediators panel under different UHPLC-MS/MS chromatography conditions (pdf)’, Data Citation 1.

As previously suggested, the elution behavior of a mixture of all compounds was first examined in the mobile phase at pH of 3.8 (ref. 16). However, since most of the standard lipids were differentiated either through MRM^HR^ transition or their respective chromatographic retention times, this analytical method could not be used to quantify lipids like LTC_4_, AA, and EPA under these conditions. As such, no chromatographic peaks were detected for the aforementioned lipids. Moreover, the shape of the peak obtained for 6-keto-PGF1α was too broad. Given this, a range of pH levels was evaluated using the developed LC method in order to optimize sensitivity and selectivity. At pH 6.4, the analytical response for all lipid species was improved, including those for LTC_4_, AA, and EPA. It is theorized that, at this pH, the lipid mediators that contained free carboxyl moieties were abundant and present as the negative [M−H]^−^ species. On the other hand, isomers with identical MRM^HR^ channels were co-eluted with the same retention time under these LC conditions. Using a mobile phase with pH of 5.8 could resolved these critical co-elution isomers while still enabling the detection of LTC_4_, AA, and EPA. As evidence of this, it was noted that the peak shape of 6-keto-PGF_1α_ was improved under these conditions (Fig. 3).

### Validity of the UHPLC-MS/MS method

Validation assays of the UHPLC-MS/MS method were attained through analysis of non-endogenous lipid mediator standards diluted to various concentrations. Reference standards were quantified as described in the data processing section. UHPLC-MS/MS method validation showed that accuracy values ranged from 0.3 to 15.0% for LOQ, from 0.4 to 13.7% for MOQ, and from 0.2 to 10.1% for HOQ on the calibration curve. Method precision for five technical replicates was between 2.3 and 14.4% for LOQ, 1.2 and 14.8% for MOQ, and between 2.1 and 14.9% for HOQ. These were in accordance with FDA guidelines for targeted LC-MS methods, which proposed a maximum CV of 15%. The dynamic range of the method covered more than four orders of magnitude and was between 0.02 and 500 ng.mL^−1^. Linear fits for the calibration curves obtained in this concentration range yielded correlation coefficients (r^2^) of up to 0.99 (Table 2), which was acceptable for a targeted lipidomics method. Furthermore, there was a measure of comparability in the IS across the whole range of retention times for equivalent mixtures of the IS and MWS. The retention time of the IS and their correspondent non-deuterated lipid standards matched exactly during the course of the LC runs. Table 2 shows the linearity and LLOQ for each lipid mediator as determined at pH 5.8 alongside their corresponding chromatographic retention time. Precision and accuracy values are shown in Table 3 (available online only). Matrix effect and recoveries were determined for the deuterated analytes since it was thought that the biological samples studied contained endogenous lipid mediators that would interfere with the experiment. The recoveries and matrix effect data obtained for plasma, lung tissue and culture medium samples are given in Tables 4, 5 and 6, respectively. For PGE_2_-*d*_*4*_, the recovery of extraction process in plasma was 74.7% when the HCQ concentration was used for spiking the biological fluid; 91.7% for MHC and 94.6% for LQC. For lung tissue we observed a recovery of 53.9% for HQC, 64.6% for MQC and 60.5% for LQC. In cell culture the recovery was 56.6% for HCQ, 78.8% for MQC and 70.5% for LC. Thus, considering all analytes the recoveries ranged from 27 to 136% for the plasma samples, from 12 to 92% for the lung tissues samples, and 34 to 79% for the culture medium, approximately. The matrix effect results observed for the samples of plasma, lung tissue and culture medium ranged, approximately, from 26 to 138%, from 23 to 145%, and from 38 to 126%, respectively.

## Usage Notes

The eicosanoids data file acquired by the TripleTOF5600^+^ instrument are publicly available in ‘.wiff’ file format at European Bioinformatics Institute’s MetaboLights repository (Data Citation 2). After download, the data can be available for analysis by different software packages. For this, Sciex (Foster, CA, USA) offer a tool called MS Data Converter, free download at https://sciex.com/software-support/software-downloads. Output can be a simple MGF peak list or an mzML file containing a fairly complete representation of the raw data. Choosing an exact translation of the instrument recorded or converts to a processed version, reducing the data down to peak lists. The mzML format is the single XML standard mass spectrometry format that was created by the merger of the older mzXML and mzData formats^20^. However, the conversion of data in mzML output files result in larger files than the original raw data^20^. To require mzXML format than mzML, it was recommended to first convert to mzML using the MS Data Converter and then use a public tool such as msconvert in the ProteoWizard package to convert mzML to mzXML, avaible in http://proteowizard.sourceforge.net/downloads.shtml. Informatics challenge associated with analysing MS data required a wide variety of data file formats to encode the complex data types associated with MS workflows^20^. Each company has developed individual formats of its own and continually extends them, as emerging instrumentation requires new features^20^. Sciex (Foster, CA, USA) instruments are saved as files with the ‘.wiff’ extension. These files might sometimes contain all information in a run or alternatively might contain only metadata and be paired with a file having ‘.wiffscan’ extension that contains the spectra^20^. To solution this, the new format mzML were created that would include the best features from both mzXML and mzData and eventually replace them^20^. Likely the most common text format is the Mascot Generic Format (MGF) file. It is still common convert the binary mass spectrometer output files into simple text files containing only the MS/MS spectra^20^. Finally, targeted and quantitative data obtained by UHPLC-MS/MS method technology in this data descriptor can be automatically processed and rapid evaluation. Also, the data sets documented her will allow draught subsets of lipid species suggestive for a physiological and metabolic condition.

## Additional information

**How to cite this article**: Sorgi, C. A. *et al*. Comprehensive High-Resolution Multiple-Reaction Monitoring Mass Spectrometry for Targeted Eicosanoid Assays. *Sci. Data* 5:180167 doi: 10.1038/sdata.2018.167 (2018).

**Publisher’s note**: Springer Nature remains neutral with regard to jurisdictional claims in published maps and institutional affiliations.

## Supplementary Material



## Figures and Tables

**Table 1:** MRM^HR^ channel parameters used for quantification of the lipid mediators via LC-MS/MS.

**Common Name**	**IS**	**DP (V)**	**CE (±2V)**	**Precursor** ***m/z***	**Product** ***m/z***
LTC_4_	LTC_4_-*d*_*5*_	-23	-40	624.2969	272.0893
LTD_4_	LTD_4_-*d*_*5*_	-30	-29	495.2531	143.0464
LTE_4_	LTE_4_-*d*_*5*_	-30	-30	438.2312	235.1529
RvD_2_	RvD_1_-*d*_*4*_	-23	-29	375.2170	141.0538
6-keto-PGF_1α_	6-keto-PGF_1α_-*d*_*4*_	-26	-24	369.2274	163.1132
PGG_2_	PGE_2_-*d*_*4*_	-27	-28	367.2121	235.0903
10(S),17(S)-diHDoHE	RvD_1_-*d*_*4*_	-23	-26	359.2222	153.0920
PGF_2α_	PGF_2α_-*d*_*4*_	-28	-38	353.2326	309.2179
PGE_2_	PGE_2_-*d*_*4*_	-27	-25	351.2168	189.1294
PGD_2_	PGD_2_-*d*_*4*_	-31	-25	351.2168	189.1295
15-keto-PGE_2_	PGE_2_-*d*_*4*_	-23	-28	349.2017	287.1995
11,12-diHETrE	5-HETE-*d*_*8*_	-37	-29	337.2377	167.1086
LTB_4_	12-*epi*-LTB_4_-*d*_*4*_	-25	-28	335.2221	129.0552
PGJ_2_	PGD_2_-*d*_*4*_	-23	-23	333.2065	189.1290
11-HETE	12-HETE-*d*_*8*_	-25	-29	319.2272	167.1078
5-oxo-ETE	5-oxo-ETE-*d*_*7*_	-23	-33	317.2116	203.1825
15-deoxi-δ12,14-PGJ_2_	15-deoxi-δ-12,14-PGJ_2_-*d*_*4*_	-24	-31	315.1962	203.1422
AA	AA-*d*_*8*_	-22	-31	303.2324	259.2447
EPA	AA-*d*_*8*_	-32	-30	301.2172	257.2306
11-*trans*-LTD_4_	LTD_4_-*d*_*5*_	-30	-30	495.2620	143.0468
TXB_2_	TXB_2_-*d*_*4*_	-22	-24	369.2277	169.0874
20-OH-PGE_2_	PGE_2_-*d*_*4*_	-30	-31	367.2119	287.1995
7(R)-Maresin-1	RvD_1_-*d*_*4*_	-30	-27	359.2221	250.1228
19-OH-PGB_2_	PGE_2_-*d*_*4*_	-28	-39	349.2015	175.1131
14,15-diHETrE	5-HETE-*d*_*8*_	-22	-29	337.2377	207.1393
5,6-diHETE	5-HETE-*d*_*8*_	-35	-25	335.2211	115.0403
PGB_2_	PGE_2_-*d*_*4*_	-27	-32	333.2067	175.1125
5-HETE	5-HETE-*d*_*8*_	-22	-31	319.2275	115.0388
12-oxo-ETE	5-oxo-ETE-*d*_*7*_	-35	-30	317.2116	153.1300
20-OH-LTB_4_	12-*epi*-LTB_4_-*d*_*4*_	-27	-30	351.2195	129.0544
6-*trans*-LTB_4_	12-*epi*-LTB_4_-*d*_*4*_	-20	-27	335.2248	129.0557
12-HETE	12-HETE-*d*_*8*_	-27	-30	319.2289	179.1071
15-oxo-ETE	5-oxo-ETE-*d*_*7*_	-15	-28	317.2145	113.0979
5S,6R-LXA_4_	5S,6R-LXA_4_-*d*_*5*_	-22	-25	351.2192	217.1598
8-HETE	5-HETE-*d*_*8*_	-22	-30	319.2297	155.0720
PGH_2_	PGE_2_-*d*_*4*_	-29	-24	351.2186	271.2051
12-HETE	12-HETE-*d*_*8*_	-27	-30	319.2291	179.1081
20-HETE	15-HETE-*d*_*8*_	-18	-34	319.2293	289.2165
15-HETE	15-HETE-*d*_*8*_	-33	-30	319.2351	175.1496
5,6-DiHETrE	5-HETE-*d*_*8*_	-27	-31	337.2427	145.0513
6-keto-PGF_1α_-*d*_*4*_	-	-35	-30	373.2534	211.1287
TXB_2_-*d*_*4*_	-	-24	-19	373.2524	173.1132
PGE_2_-*d*_*4*_	-	-37	-23	355.2424	275.2333
PGD_2_-*d*_*4*_	-	-46	-18	355.2424	237.1451
15-deoxi-δ-12,14-PGJ_2_-*d*_*4*_	-	-27	-23	319.2216	275.2341
PGF_2α_-*d*_*4*_	-	-37	-39	357.2583	177.1486
LTB_4_-*d*_*4*_	-	-29	-43	339.2479	183.0386
5-HETE-*d*_*8*_	-	-24	-25	327.2791	116.0473
12-HETE-*d*_*8*_	-	-34	-25	327.2791	184.1390
15-HETE-*d*_*8*_	-	-47	-27	327.2791	226.1840
5-oxo-ETE-*d*_*7*_	-	-29	-32	324.2554	210.2230
RvD_1_-*d*_*4*_	-	-24	-27	350.2491	220.1764
LTC_4_-*d*_*5*_	-	-20	-39	629.3274	272.0899
LTE_4_-*d*_*5*_	-	-34	-27	443.2634	338.2218
AA-*d*_*8*_	-	-35	-36	311.2832	237.0907
LTD_4_-*d*_*5*_	-	-26	-28	500.2848	482.2772
12-*epi*-LTB_4_-*d*_*4*_	-	-36	-26	339.2471	197.1152
5S,6R-LXA_4_-*d*_*5*_	-	-30	-28	356.2491	222.1907
*Window of 0.05 Da					

**Table 2:** Retention time, r^2^, and LLOQ for all lipid mediator targets analyzed at pH 5.8.

**Common name**	**Retention time (min)**	***r***^***2***^	**LLOQ**^a^**(pg)**
20-OH-LTB_4_	3.06	0.9986	0.05
LTC_4_	5.09	0.9987	0.09
PGB_2_	8.70	0.9993	0.02
15-keto-PGE_2_	5.99	0.9994	0.05
20-OH-PGE_2_	1.80	0.9989	0.05
TXB_2_	4.06	0.9987	0.05
LXA_4_	5.93	0.9993	0.09
PGD_2_	5.56	0.9988	0.05
6-keto-PGF_1α_	2.45	0.9963	0.18
PGE_2_	5.20	0.9993	0.02
RvD_2_	6.23	0.9989	0.09
PGF_2α_	4.63	0.9968	0.09
19-OH-PGB_2_	2.28	0.9982	0.09
PGG_2_	6.73	0.9919	0.37
LTB_4_	10.54	0.9981	0.02
LTD_4_	6.90	0.9993	0.05
LTE_4_	7.90	0.9987	0.09
6-*trans*-LTB_4_	10.09	0.9977	0.05
11-*trans*-LTD_4_	6.90	0.9991	0.09
PDx	10.40	0.9991	0.05
MaR-1	10.21	0.9986	0.05
PGH_2_	7.61	0.9966	0.09
PGJ_2_	8.65	0.9993	0.05
15-deoxy-δ-12,14-PGJ_2_	13.23	0.9970	0.09
5-HETE	14.41	0.9989	0.05
AA	17.17	0.9675	0.09
5-oxo-ETE	14.68	0.9986	0.02
20-HETE	13.35	0.9977	0.09
5,6-DiHETE	12.91	0.9992	0.02
12-HETE	14.22	0.9993	0.05
8-HETE	14.22	0.9984	0.02
11-HETE	14.08	0.9972	0.05
12-oxo-ETE	14.69	0.9980	0.05
15-oxo-ETE	13.99	0.9969	0.05
11,12-DiHETrE	12.94	0.9981	0.05
14,15-DiHETrE	12.73	0.9931	0.05
EPA	16.71	0.9951	0.09
5,6-DiHETrE	13.22	0.9974	0.09
15-HETE	13.89	0.9967	0.09
* Calibration curves include six runs, with ten concentrations.			

^a^Lower limit of quantification values were considered for accuracy within 20% of the nominal concentration (CV ≤20%).

**Table 3:** Quality control analysis of lipid mediators to evaluate the precision and accuracy of the MRM^HR^ method.

**Analytes**		**Intraday (*****n***=**5)** a		**Interday (3 days)** b	
		**RSD %** c	**RE %** d	**RSD %** c	**RE %** d
20-OH-LTB_4_	LQC	16.44	11.39	11.08	4.44
	MQC	5.98	6.90	12.90	8.67
	HQC	4.07	3.36	7.05	1.04
LTC_4_	LQC	10.49	7.78	--	--
	MQC	5.62	7.67	14.37	5.07
	HQC	4.38	0.77	14.48	3.66
PGB_2_	LQC	5.93	0.56	12.73	19.44
	MQC	3.47	1.33	8.72	4.53
	HQC	5.31	4.01	7.60	2.98
15-keto-PGE_2_	LQC	11.38	1.39	11.03	1.11
	MQC	5.24	0.37	9.12	4.20
	HQC	4.84	2.61	5.06	2.48
20-OH-PGE_2_	LQC	9.95	2.50	19.11	3.33
	MQC	5.62	6.27	9.69	0.47
	HQC	5.31	3.31	7.88	4.86
TXB_2_	LQC	8.32	4.72	11.26	2.78
	MQC	7.53	4.57	8.40	4.13
	HQC	5.36	4.29	5.04	3.76
LXA_4_	LQC	17.74	6.39	11.28	8.89
	MQC	7.93	2.17	7.47	1.47
	HQC	1.63	0.97	4.26	0.20
PGD_2_	LQC	6.58	0.56	10.53	4.44
	MQC	4.11	3.27	4.09	3.73
	HQC	4.16	0.84	4.73	3.78
6-keto-PGF_1α_	LQC	11.07	5.00	19.10	15.00
	MQC	10.59	10.10	7.89	2.27
	HQC	8.46	10.14	8.88	5.90
PGE_2_	LQC	5.99	9.28	5.95	0.01
	MQC	1.25	3.43	1.63	0.93
	HQC	3.76	2.13	7.35	1.08
RvD_2_	LQC	10.64	5.83	6.56	3.33
	MQC	9.69	0.77	7.19	0.77
	HQC	2.55	5.27	6.97	8.96
PGF_2α_	LQC	8.35	12.78	--	--
	MQC	9.65	4.63	15.00	4.31
	HQC	2.33	0.43	2.99	1.38
19-OH-PGB_2_	LQC	8.80	11.11	12.71	13.33
	MQC	7.06	4.73	12.13	10.60
	HQC	7.14	8.72	8.30	2.40
PGG_2_	LQC	--	--	--	--
	MQC	8.88	4.00	--	--
	HQC	13.70	2.93	--	--
LTB_4_	LQC	10.83	10.00	14.76	6.11
	MQC	5.23	1.77	8.43	6.53
	HQC	2.96	4.70	2.86	1.94
LTD_4_	LQC	12.34	1.39	--	--
	MQC	5.20	0.83	8.03	12.53
	HQC	2.73	0.57	5.06	6.34
LTE_4_	LQC	8.88	3.33	--	--
	MQC	7.54	0.93	9.68	5.27
	HQC	7.70	7.28	5.08	0.18
6-*trans*-LTB_4_	LQC	3.16	7.22	--	--
	MQC	7.46	6.93	3.90	0.60
	HQC	5.13	2.21	7.54	3.80
11-*trans*-LTD_4_	LQC	7.96	0.01	8.86	10.56
	MQC	2.77	0.50	7.43	0.40
	HQC	2.93	2.95	8.54	5.52
PDx	LQC	5.17	3.89	7.04	1.11
	MQC	5.57	1.93	3.66	4.00
	HQC	4.54	1.75	4.37	1.32
MaR-1	LQC	8.79	1.67	--	--
	MQC	7.27	0.80	10.83	4.80
	HQC	5.13	3.00	8.58	0.32
PGH_2_	LQC	14.41	7.50	--	--
	MQC	7.91	6.30	13.56	6.33
	HQC	5.17	0.27	7.87	3.78
PGJ_2_	LQC	6.28	8.06	13.74	9.44
	MQC	5.02	4.47	9.16	9.27
	HQC	3.51	0.40	4.34	2.34
15-deoxy-δ-12,14-PGJ_2_	LQC	6.00	7.78	--	--
	MQC	4.78	3.40	3.27	2.53
	HQC	3.80	5.95	3.73	4.98
5-HETE	LQC	9.68	5.28	8.13	7.78
	MQC	4.24	4.70	3.33	8.87
	HQC	4.45	2.28	2.92	6.38
AA	LQC	8.02	5.00	--	--
	MQC	14.82	0.70	--	--
	HQC	--	--	14.95	0.22
5-oxo-ETE	LQC	6.15	1.94	--	--
	MQC	2.47	1.80	12.98	11.27
	HQC	3.29	1.23	2.07	6.30
20-HETE	LQC	2.72	9.17	--	--
	MQC	5.04	0.53	5.82	1.87
	HQC	4.19	2.53	9.49	5.82
5,6-DiHETE	LQC	6.46	1.94	12.68	16.67
	MQC	2.51	6.40	4.51	3.13
	HQC	3.32	1.02	2.87	0.98
12-HETE	LQC	4.43	2.22	11.41	3.33
	MQC	4.93	4.13	6.05	6.87
	HQC	3.85	4.83	3.26	1.28
8-HETE	LQC	4.50	1.39	14.26	1.67
	MQC	6.17	4.13	4.67	2.27
	HQC	3.87	1.00	3.63	2.74
11-HETE	LQC	4.68	9.17	8.61	10.00
	MQC	1.27	5.60	3.51	1.13
	HQC	6.05	8.69	2.69	0.36
12-oxo-ETE	LQC	7.45	2.50	17.21	8.89
	MQC	4.12	5.33	4.71	1.40
	HQC	3.93	3.94	9.52	1.52
15-oxo-ETE	LQC	2.35	7.78	9.31	1.67
	MQC	3.43	6.20	5.44	1.47
	HQC	2.83	6.33	4.87	1.74
11,12-DiHETrE	LQC	4.84	0.56	9.19	11.11
	MQC	2.09	8.87	2.41	2.40
	HQC	3.64	6.11	1.92	0.60
14,15-DiHETrE	LQC	3.09	0.83	12.46	12.78
	MQC	6.94	7.07	7.82	13.73
	HQC	7.28	9.71	4.66	3.60
EPA	LQC	8.52	5.00	--	--
	MQC	8.60	0.67	11.25	12.80
	HQC	4.33	5.25	5.20	2.54
5,6-DiHETrE	LQC	3.13	5.28	15.91	0.28
	MQC	2.37	4.07	3.26	4.80
	HQC	2.02	2.29	2.46	3.06
15-HETE	LQC	8.75	0.28	12.91	8.89
	MQC	9.10	3.13	8.07	1.30
	HQC	5.44	8.51	5.30	0.52

^a^Number of measurements

^b^number of days

^c^relative standard deviation, in percentage points (%)

^d^relative error in percentage points (%).

**Table 4:** Mean recovery and matrix effect of the lipid mediator targets using SPE extraction in HQC, MQC, and LQC levels of IS in plasma samples.

**Internal Standard**	**Recovery Rate (%**±**SD)**^a^			**Matrix Effect (%± SD)**^b^		
	**HQC**^c^	**MQC**^d^	**LQC**^e^	**HQC**^c^	**MQC**^d^	**LQC**^e^
15-HETE-*d*_*8*_	80.5±12.1	99.6±11.7	135.2±25.4	106.4±7.6	118.8±20.0	139.7±11.6
TXB_2_-*d*_*5*_	59.9±7.9	70.4±6.8	75.3±11.1	70.9±3.9	72.1±9.18	70.4±7.1
6-keto-PGF_1__-*d*_*4*_	136.2±15.1	145.8±25.3	------	145.3±14.9	154.4±27.4	-----
PGE_2_-*d*_*4*_	74.7±8.6	91.7±5.6	94.6±13.2	88.5±6.4	100.1±15.3	93.7±10.4
PGF_2__-*d*_*4*_	84.4±8.2	95.6±4.2	89.0±29.3	101.9±9.7	106.7±23.7	96.3±17.8
LXA_4_-*d*_*5*_	74.1±7.5	84.1±10.9	-----	101.8±9.2	96.8±18.8	150.1±24.4
RvD_1_-*d*_*5*_	76.1±9.1	92.0±9.4	115.1±13.7	100.6±10.2	107.2±21.4	107.0±21.9
PGD_2_-*d*_*4*_	44.8±3.6	48.0±8.2	57.6±12.7	41.3±3.4	48.5±8.5	59.5±13.7
12-*epi*-LTB_4_-*d*_*4*_	79.7±3.4	98.0±11.2	104.2±14.4	97.1±6.8	109.7±17.4	116.3±11.9
LTE_4_-*d*_*5*_	62.2±7.5	69.9±7.9	-----	132.1±6.6	141.5±24.9	201.8±102.8
AA-*d*_*8*_	51.3±1.9	27.9±4.8	21.9±5.1	58.7±5.9	27.6±6.7	26.1±6.4
15-deoxy-δ-12,14-PGJ_2_-*d*_*4*_	69.4±9.5	94.0±22.9	191.8±29.0	109.8±8.2	131.1±32.9	167.7±30.1
5-HETE-*d*_*8*_	76.8±10.4	96.7±8.7	96.6±13.4	99.1±6.5	102.2±15.4	117.7±7.4
5-oxo-ETE-*d*_*7*_	81.9±12.9	87.9±12.2	108.4±25.5	99.8±6.8	93.5±16.7	120.7±24.9
12-HETE-*d*_*8*_	78.7±11.9	99.2±8.0	126.1±14.8	104.2±8.5	121.7±21.2	138.7±14.0
*n=6, injected in triplicate.						

^a^Recovery were performed by comparing the extracted samples with unextracted standards that represent 100%.

^b^Matrix effect expressed as the ratio of the mean peak area of an analyte spiked post-extraction to the mean peak area of the same analyte standards multiplied by 100. A value of >100% indicates ionization enhancement, and a value of <100% indicates ionization suppression.

^c^High-concentration Quality Control.

^d^Medium-concentration Quality Control.

^e^Low-concentration Quality Control.

**Table 5:** Mean recovery and matrix effect of the lipid mediator targets using SPE extraction in HQC, MQC, and LQC levels of IS in lung samples.

**Internal Standard**	**Recovery Rate (%**±**SD)**^a^			**Matrix Effect (%± SD)**^b^		
	**HQC**^c^	**MQC**^d^	**LQC**^e^	**HQC**^c^	**MQC**^d^	**LQC**^e^
15-HETE-*d*_*8*_	70.4±5.7	86.8±16.9	92.9±11.9	94.1±2.7	145.1±17.4	117.5±13.9
TXB_2_-*d*_*5*_	54.8±3.72	67.2±14.6	64.1±10.1	82.4±3.5	108.6±19.8	104. 4±11.6
6-keto-PGF_1α_-*d*_*4*_	50.3±5.0	68.6±16.2	------	78.7±6.5	128.6±45.3	--------
PGE_2_-*d*_*4*_	53.9±5.1	64.6±11.8	60.5±16.2	77.1±4.1	105.0±12.6	95.8±8.7
PGF_2__-*d*_*4*_	56.3±5.6	65.8±14.9	40.6±12.9	79.1±5.8	102.1±16.1	82.1±15.3
LXA_4_-*d*_*5*_	57.0±6.3	69.2±12.2	------	78.2±4.2	99.8±8.4	168.7±54.9
RvD_1_-*d*_*5*_	53.9±6.4	63.6±12.5	68.4±15.0	74.9±3.2	103.7±10.7	106.9±18.0
PGD_2_-*d*_*4*_	48.7±3.8	58.8±13.8	88.6±7.0	73.5±4.7	99.8±12.4	135.5±14.4
12-*epi*-LTB_4_-*d*_*4*_	53.8±3.8	64.1±13.6	68.2±15.1	72.0±2.9	99.9±14.3	91.4±12.2
LTE_4_-*d*_*5*_	45.2±3.9	59.6±17.2	144.2±35.1	82.3±6.5	118.8±17.9	301.9±52.5
AA-*d*_*8*_	51.8±16.4	17.0±5.8	12.7±4.3	66.5±12.9	24.7±4.9	23.7±5.6
15-deoxy-δ-12,14-PGJ_2_-*d*_*4*_	52.1±4.2	70.9±10.4	79.8±13.2	69.2±5.2	121.1±10.1	105.4±7.4
5-HETE-*d*_*8*_	58.0±5.2	65.6±11.3	62.4±12.6	77.5±6.4	104.7±16.2	91.4±9.3
5-oxo-ETE-*d*_*7*_	67.9±9.0	61.5±8.1	50.4±26.6	89.9±6.6	111.0±16.9	83.7±12.7
12-HETE-*d*_*8*_	61.7±5.6	74.3±13.4	75.8±12.0	78.9±4.1	118.4±10.9	94.9±14.0
*n=6, injected in triplicate.						

^a^Recovery were performed by comparing the extracted samples with unextracted standards that represent 100%.

^b^Matrix effect expressed as the ratio of the mean peak area of an analyte spiked post-extraction to the mean peak area of the same analyte standards multiplied by 100. A value of >100% indicates ionization enhancement, and a value of < 100% indicates ionization suppression.

^c^High-concentration Quality Control.

^d^Medium-concentration Quality Control.

^e^Low-concentration Quality Control.

**Table 6:** Mean recovery and matrix effect of the lipid mediator targets using SPE extraction in HQC, MQC, and LQC levels of IS in cell culture medium.

**Internal Standard**	**Recovery Rate (%**±**SD)**^a^			**Matrix Effect (%± SD)**^b^		
	**HQC**^c^	**MQC**^d^	**LQC**^e^	**HQC**^c^	**MQC**^d^	**LQC**^e^
15-HETE-*d*_*8*_	42.7±5.8	56.6±12.8	52.3±8.9	79.9±12.9	114.1±3.6	118.9±9.8
TXB_2_-*d*_*5*_	58.3±6.4	58.9±2.9	58.6±5.2	54.2±6.9	74.59±4.9	62.0±10.1
6-keto-PGF_1α_-*d*_*4*_	34.4±4.5	74.9±17.1	-------	95.5±7.7	126.9±33.3	-------
PGE_2_-*d*_*4*_	56.7±3.2	78.8±5.0	71.5±4.9	64.2±12.3	94.3±3.8	80.1±10.2
PGF_2__-*d*_*4*_	73.1±7.1	82.5±8.0	74.2±19.2	78.5±17.1	104.4±11.6	79.9±20.9
LXA_4_-*d*_*5*_	44.5±5.7	51.5±4.3	-------	48.6±6.6	62.4±4.9	-------
RvD_1_-*d*_*5*_	49.9±10.1	51.9±3.7	57.6±7.1	49.2±9.4	67.4 ±5.6	63.4±13.4
PGD_2_-*d*_*4*_	49.3±8.5	54.8±3.9	56.7±10.8	47.3±8.7	69.4±2.9	73.6±9.3
12-*epi*-LTB_4_-*d*_*4*_	61.1±8.2	59.8±3.2	63.0±14.4	66.2±11.9	87.9±5.4	78.4±10.1
LTE_4_-*d*_*5*_	73.3±36.6	34.3±5.1	80.8±28.1	68.5±15.7	93.6±8.5	114.4±43.2
AA-*d*_*8*_	44.3±8.4	12.3±7.1	-------	47.2±5.3	35.4±3.5	38.7±6.5
15-deoxy-δ-12,14-PGJ_2_-*d*_*4*_	40.2±5.3	43.8±5.0	59.6±5.4	54.0±9.5	77.3±5.6	83.3±5.3
5-HETE-*d*_*8*_	37.1±4.2	46.3±12.1	35.6±13.5	79.91± 14.3	103.9±5.6	94.9±8.3
5-oxo-ETE-*d*_*7*_	41.5±9.2	36.3±13.2	-------	83.3±16.0	96.4±10.2	81.2±14.3
12-HETE-*d*_*8*_	43.5±6.8	42.4±9.6	42.3±15.2	71.5±12.5	98.9±4.6	109.5±9.8
*n=6, injected in triplicate.						

^a^Recovery were performed by comparing the extracted samples with unextracted standards that represent 100%.

^b^Matrix effect expressed as the ratio of the mean peak area of an analyte spiked post-extraction to the mean peak area of the same analyte standards multiplied by 100. A value of >100% indicates ionization enhancement, and a value of < 100% indicates ionization suppression.

^c^High-concentration Quality Control.

^d^Medium-concentration Quality Control.

^e^Low-concentration Quality Control.
